# Mechanisms of interstrand DNA crosslink repair and human disorders

**DOI:** 10.1186/s41021-016-0037-9

**Published:** 2016-05-01

**Authors:** Satoru Hashimoto, Hirofumi Anai, Katsuhiro Hanada

**Affiliations:** Department of Clinical Pharmacology and Therapeutics, Faculty of Medicine, Oita University, 1-1 Idaigaoka, Hasama-machi, Yufu, Oita 879-5593 Japan; Clinical Engineering Research Center, Faculty of Medicine, Oita University, 1-1 Idaigaoka, Hasama-machi, Yufu, Oita 879-5593 Japan

**Keywords:** Homologous recombination, Nucleotide excision repair, Translesion DNA synthesis, Fanconi anemia

## Abstract

Interstrand DNA crosslinks (ICLs) are the link between Watson-Crick strands of DNAs with the covalent bond and prevent separation of DNA strands. Since the ICL lesion affects both strands of the DNA, the ICL repair is not simple. So far, nucleotide excision repair (NER), structure-specific endonucleases, translesion DNA synthesis (TLS), homologous recombination (HR), and factors responsible for Fanconi anemia (FA) are identified to be involved in ICL repair. Since the presence of ICL lesions causes severe defects in transcription and DNA replication, mutations in these DNA repair pathways give rise to a various hereditary disorders. NER plays an important role for the ICL recognition and removal in quiescent cells, and defects of NER causes congential progeria syndrome, such as xeroderma pigmentosum, Cockayne syndrome, and trichothiodystrophy. On the other hand, the ICL repair in S phase requires more complicated orchestration of multiple factors, including structure-specific endonucleases, and TLS, and HR. Disturbed this ICL repair orchestration in S phase causes genome instability resulting a cancer prone disease, Fanconi anemia. So far more than 30 factors in ICL repair have already identified. Recently, a new factor, UHRF1, was discovered as a sensor of ICLs. In addition to this, numbers of nucleases that are involved in the first incision, also called unhooking, of ICL lesions have also been identified. Here we summarize the recent studies of ICL associated disorders and repair mechanism, with emphasis in the first incision of ICLs.

## Background

Interstrand DNA crosslinks (ICLs) are lesions that are a covalent linkage between opposite strands of double-stranded DNA. They are formed in the presence of bifunctional alkylating agents [1–4]. Organisms are exposed to bifunctional alkylating agents, also called ICL-causing agents, as a result of endogenous metabolic processes as well as by exogenous stresses from environmental mutagens [3–5]. ICLs are extremely cytotoxic, as even a single ICL in the genome can cause severe defects in a variety of vital DNA metabolic processes, such as transcription and DNA replication [6, 7]. Particularly, the selective inhibitory effect of ICL agents on DNA replication—crucial for proliferation and cell survival—is used in both chemotherapy and phototherapy to treat various cancers and skin diseases [8]. On the other hand, the defect of ICL repair causes chromosome instability syndromes, such as Fanconi anemia. Recently, many new factors involved in ICL repair were identified from genetic studies of Fanconi anemia, and these studies suggested that ICL repair is performed in quite complicated mechanisms. In this review, we briefly summarize the recent studies of ICL associated disorders and repair mechanism, with emphasis in the first incision of ICLs.

## Typical ICL lesions

Chemical structures of ICL lesions have been comprehensively reviewed by Guainazzi and Schärer, and by Legerski [2, 3] and will not be discussed in detail in this review. Cisplatin and its derivatives, carboplatin and oxaliplatin, are widely used in clinical applications and can be applied to a wide variety of cancers. Cisplatin targets guanine bases in DNA, and ICLs occur at 5ʹ-GC-3ʹ sites in double-stranded DNA. The ICL formed by cisplatin shows the largest distortion of the DNA strands, compared to other ICL formed by agents described below, and it distortion is 45° of bending and 79° of unwinding [9]. Cisplatin creates not only ICL but also an intra-strand crosslink at 5′-GG-3′ sites. Similar to other bulky adducts which affect only one strand of the double-helix structures, intra-strand crosslinks caused by cisplatin are repaired by NER.

Nitrogen mustard and its derivatives also react with guanine bases, and ICL formation occurs at 5ʹ-GNC-3ʹ sites in double-stranded DNA [10]. Historically, nitrogen mustard was the first DNA damaging agent used for chemotherapy [11]. ICLs formed by nitrogen mustard show strand distortions with 14° of bending [10]. As ICL formation by nitrogen mustard is rapid (it occurs within 20 minutes of treatment), nitrogen mustard can be used in ICL repair studies in yeast [12]. Psoralen and its derivatives can form ICLs following activation with long wavelength ultraviolet radiation. Psoralen was isolated from *Ammi majus.* In Egypt, *Ammi majus* would be used in phototherapy during several millennia for treatment of psoriasis and leukoderma vulgaris [13]. Psoralen reacts with thymine bases, and ICLs occur at both 5ʹ-AT-3ʹ and 5ʹ-TA-3ʹ sites in double-stranded DNA. The ICL formed by psoralen induces 25° of unwinding and a minor local distortion of helical structure [14, 15]. As ICLs formed by psoralen are relatively stable in solution, it is often used in biochemical and cell biological studies. Mitomycin C is widely used for cell biological studies of ICL repair. Mitomycin C reacts with the guanine base in the minor groove of double-stranded DNA, and ICL formation occurs at 5ʹ-CG-3ʹ sites. The ICL formed by mitomycin C does not significantly distort the double helix [16, 17].

## ICL removal in quiescent G0/G1 phase cells

**Fig. 1:**
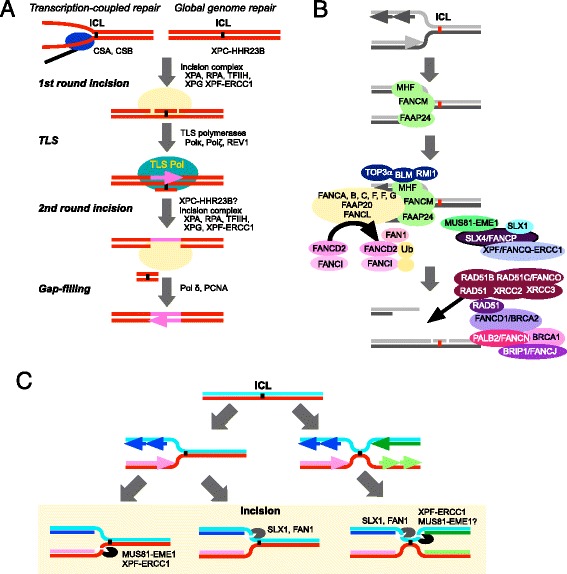
Models of ICL repair. **a** Model of ICL repair in quiescent cells (G0/G1 phase). An ICL on DNA is recognized by NER machinery. In the case of ICL-blocked transcription, two specific factors for transcription-coupled NER, CSA and CSB, are required to load the incision complex. In contrast, for ICLs in non-transcribed regions, the XPC-HHR23B complex is responsible for loading of incision complex of NER. The first incision is introduced by the incision complex composed of XPA-RPA, TFIIH, XPF-ERCC1 and XPG. After the first incision, the ICL lesion with the oligonucleotide is bypassed by a TLS polymerase such as DNA polymerase κ, DNA polymerase ζ, or REV1. The second incision is the introduced by another NER incision complex. **b** Model of ICL repair in S phase. ICL lesions cause stalling of DNA replication forks. The FANCM-FAAP24-MHF complex binds to a stalled replication fork and recruits both the FA core complex and the BLM-TOP3α-RMI1 complex. Activated FA core complex mono-ubiquitinates both FANCD2 and FANCI, which permits incisions of the ICL using structure-specific endonucleases such as XPF/FANCQ-ERCC1, SLX4/FANCP-SLX1, MUS81-EME1 and FAN1. The incision introduces a DSB which is repaired by. HR. Both RAD51 paralogs (RAD51B, RAD51C/FANCO, RAD51D, XRCC2 and XRCC3) and BRCA complexes (BRCA1, BRCA2/FANCD1, PALB2/FANCN, and BRIP1/FANCJ) are required for the formation of RAD51 filaments at damage sites. **c** Models of ICL incisions. An ICL lesion causes a stalled DNA replication fork that must be resolved by ICL incision. Three models for this process have been suggested. One model suggests that the first incision involves cleavage of the leading strand at a single stalled replication fork. The second model suggests that the first incision involves cleavage of the lagging strand at a single stalled replication fork. The third model suggests cleavage at two converged replication forks. After incision, the oligonucleotide with an ICL lesion is bypassed by a TLS polymerase, such as DNA polymerase κ, DNA polymerase ζ, or REV1, The DSB end is subsequently repaired by homologous recombination

Understanding the molecular mechanisms of ICL repair is exceptionally challenging because an ICL lesion affects both DNA strands. When a DNA lesion is located on only one DNA strand, the DNA fragment with lesion is excised by the introduction of two single-stranded breaks on either side of the lesion. This principle is common among various excision repair pathways, including nucleotide excision repair (NER), base excision repair (BER), and mismatch repair (MMR). However, in the case of ICLs, repair mechanisms involving a simple excision followed by template resynthesis are not sufficient [18]. In quiescent cells (cells in G0/G1 phase), HR is not essential for ICL repair [19]. Therefore, in all eukaryotes from *Saccharomyces cerevisiae* to humans, both the first and second rounds of ICL incisions occur by NER (Fig. 1a) [19, 20]. The ICL lesion with the oligonucleotide on the single-stranded gap produced by the first round of NER is bypassed with translesion DNA polymerases, such as DNA polymerases η, ι, κ, and ζ, and REV1 (Fig. 1) [19, 21–23]. In particular, DNA polymerases κ, and ζ, and REV1 seem to be important for this step [19, 21, 22, 24].

## ICL recognition in proliferating S phase cells

The process of ICL repair in S phase is quite complicated. Many studies have observed that treatment with ICL-causing agents introduces double-stranded DNA breaks (DSBs) in S phase cells [7, 25, 26]. One curious phenomenon about ICL-induced DSBs is that they are repaired by HR and not by non-homologous end joining (NHEJ) [12, 25]. Such phenomena strongly indicate that ICL-induced DSBs are associated with DNA replication forks. In *S. cerevisiae*, ICLs are mostly recognized by NER and complete NER function is responsible for the incisions. Therefore, all NER-mutants show hyper sensitive to ICL agents [25, 27]. In contrast, only *XPF*- and *ERCC1*-deficient cells are extremely hypersensitive to ICL agents, such as mitomycin C and nitrogen mustard in mammalian cells [25, 27]. The gene products of *XPF* and *ERCC1* form a hetero-dimeric endonuclease that specifically recognizes and cleaves single-stranded branched structures [28]. Interestingly, the homologous structure-specific endonucleases MUS81-EME1 and XPF-ERCC1, are also involved in the repair process of ICL removal [7, 29]. MUS81-EME1 preferentially binds double-stranded branched structures, 3ʹ-flaps, and Holliday junctions [30]. Both XPF-ERCC1 and MUS81-EME1 are involved in ICL-induced DSB formation. However, further investigation is required to confirm whether DSB formation is directly involved in the removal of ICLs (Fig. 1b) [7, 31]. As many nucleases involved in the incision of ICLs have been recently identified, understanding the mechanisms of ICL incision is relevant for DNA repair. Here, we summarise our current understanding of ICL repair mechanisms in S phase. DSBs induced by ICLs in S phase are repaired by HR. In *S. cerevisiae*, hypersensitivity to ICL-causing agents is observed in *rad51*, *rad52, rad54*, *rad59*, and *mre11* mutants, but not in *yku70* mutants, and the hypersensitivity of *rad52 yku70* double mutants to ICLs is comparable to that of *rad52* mutants [12]. In fact, increased accumulation of DSBs after treatment with ICL-causing agents and defects in DSB repair are observed in HR-deficient strains, suggesting that NHEJ is not required for the repair of DSBs induced by ICLs [12]. A similar phenomenon is observed in mammalian cells [25]. Hypersensitivity to ICLs is seen in HR-deficient cells, such as cells carrying mutations in *RAD51* paralogs, *RAD54*, *RAD54B*, and *BRCA2*, but not in NHEJ-deficient cells [32–34]. It is likely that HR plays a role in not only repairing DSBs but also in restarting stalled DNA replication forks [7, 35]. In higher eukaryotes, genes responsible for Fanconi anemia (FA) play important roles in ICL repair. Although the biological roles of FA gene products are not entirely characterized [36], they are known to control HR at DNA replication forks [4]. We will describe the roles of FA gene products in the next section, emphasizing the regulation of HR at stalled DNA replication forks.

## ICL repair genes and human disorders

Proteins implicated in the repair of ICLs have a critical role in the pathophysiology of several hereditary disorders, known as FA, xeroderma pigmentosum (XP), Cockayne syndrome (CS), cerebro-oculo-facio-skeletal syndrome (COFS), and trichothyodistrophy (TTD, Table 1) [37]. FA is a genetic disorder characterized by aplastic anaemia, bone marrow failure, and cancers (typically acute myelogenous leukemia) [38, 39]. Mutations in one of the *FANC* genes cause severe sensitivity to ICL agents and genomic instability [38]. So far, at least 18 genes have been implicated in FA, and all the genes products act on the ICLs repair in S phase [4, 40]. On the other side, defects in NER pathways, which have a role in G0/G1 phase, result in also rare autosomal-recessive diseases, XP, CS, COFS syndrome, and TTD [41]. Mutations in eleven genes have been associated with these NER disorders [41]. Characteristics of XP include a photosensitivity, pigmentation, and frequent skin cancers. CS is an inherited syndrome characterized by short stature, mental deficiency, photosensitivity, disproportionately large hands, feet, and ears, ocular defects, and extensive demyelination [37]. CS has wide spectrum clinical features, and the most severely affected patients are included in a category of COFS syndrome [42]. TTD has a distinct sulfur-deficient brittle hair and neuroectodermal symptoms [41, 43, 44]. These NER disorders are distinguished from each other by these physical characteristics, including cutaneous malignancies (Table 2). Interestingly only *ERCC1* and *XPF* gene products play a role in both S phase and G0/G1 phase pathways.

**Table 1:** Molecular function of ICL repair factors linked to human disorders

Gene (also known as)	Biochemical functions	Disorders	References
*FANCA*	FA core complex	FA	[57]
*FANCB*	FA core complex	FA	[58]
*FANCC*	FA core complex	FA	[59]
*FANCD1 (BRCA2)*	HR	FA, HBOC	[60]
*FANCD2*	FAN1 recruitment	FA	[61]
*FANCE*	FA core complex	FA	[62, 63]
*FANCF*	FA core complex	FA	[64]
*FANCG*	FA core complex	FA	[65]
*FANCI*	FAN1 recruitment	FA	[66, 67]
*FANCJ (BRIP1)*	HR, Chromatin remodeling factor	FA, HBOC	[68, 69]
*FANCL*	Ubiquitin ligase	FA	[70]
*FANCN (PALB2)*	HR	FA, HBOC	[71, 72]
*FANCO (RAD51C)*	HR	FA, HBOC	[73, 74]
*FANCP (SLX4)*	Structure-specific endonuclease	FA	[75]
*FANCQ (XPF)*	NER, Structure-specific endonuclease	FA, XP, CS, COFS	[76–78]
*FANCS (BRCA1)*	HR, Chromatin remodeling factor	FA, HBOC	[79]
*FANCT (UBE2T)*	E2 ubiquitin conjugating enzyme	FA	[80]
*ERCC1*	NER, Structure-specific endonuclease	COFS	[81]
*XPA*	NER	XP	[82]
*XPB*	NER, Helicase in TFIIH	XP, CS, TTD	[83–85]
*XPC*	NER	XP	[86]
*XPD*	NER, Helicase in TFIIH	XP, CS, TTD, COFS	[87–89]
*XPE*	NER	XP	[90]
*XPG*	NER	XP, CS	[91, 92]
*CSA*	NER	CS	[93]
*CSB*	NER	CS, COFS	[94]
*TTDA (p8)*	NER, a component of TFIIH	TTD	[95]

*HR* factor in homologous recombination, *NER* factor in nucleotide excision repair

*HBOC* Hereditary breast and/or ovary cancer syndrome

*FA* Fanconi anemia, *COFS* Cerebro-oculo-facio-skeletal syndrome

*XP* Xeroderma pigmentosum, *CS* Cockayne syndrome, *TTD* Trichothiodystropy

**Table 2:** Clinical features of FA, XP, CS, and TTD

Clinical features	FA	XP	CS	TTD
Cancer	+	+	-	-
Skin pigmentation	+	+	-	±
Developmental delay	+	-	+	+
Neurological defects	±	±	+	+

+ represents that this symptoms appears on almost all patients

- represents that this symptoms hardly recognized

± represents that this symptom is occasionally recognized

Given the established role of DNA repair factors as a genome keeper against a mutagenesis, it is not surprising that some of ICL genes have a strong linkage with cancer. Recent next-generation sequencing revealed the hereditary breast and/or ovary cancer syndrome (HBOC) related genes, and *BRCA1, BRCA2, BRIP1, PALB2, and RAD51C* genes are associated with HBOC in ICL repair pathways [4]. From the view of preventive medicine, early detection strategy is required in the social framework. Especially in the patients having the mutations in *BRCA1* and *BRCA2* genes, reasonable interventions are strongly recommended.

Many DNA cross-liker agents, such as cisplatin, psoralen, mitomycin C, and so on, were investigated in the long history of ICL repair fields. However none of these agents are produced in mammal internal organs. What is the pathophysiological accelerator of ICL repair defected patients? Recent study suggested the interesting story that aldehyde, one of the endogenous reactive metabolites, at least partially leads to genotoxic of FA patients [45]. Of course it is forbidden to forget that careful choice of medicine could prevent the incidental adverse event on these patients, who are apt to be given an anti cancer agents, such as cisplatin.

## ICL incisions at stalled DNA replication sites

As an ICL lesion inhibits the unwinding of DNA strands, the progression of replicative DNA helicases are completely blocked at the site of the lesion. Previously, two distinct models of incision were proposed to explain stalling of a single replication fork [26]. One model suggests that the first incision cleaves the leading strand [46]. In this case, cleavage of either the 3ʹ-end of splayed arms or the 3ʹ-flap structure is required and XPF-ERCC1 and MUS81-EME1 are potentially involved in the process (Fig. 1c) [31]. The major shortcoming of this mechanism is the loss of MCM proteins that act as replicative DNA helicases. Another model is that the first incision occurs on the lagging strand [46]. This also requires cleavage of either the 3ʹ-end of splayed arms or the 3ʹ-flap structure. SLX1 and FAN1 may be the endonucleases involved in this process (Fig. 1c). This cleavage requires reloading of RNA primase-DNA polα complexes to restart DNA replication. Loading of MCM2-7 and RNA primase-DNA polα complexes at origins of DNA replication is strictly regulated in eukaryotic cells. Although it was suggested that MCM8-9 is involved in ICL repair to promote HR, the mechanism through which the complete DNA replication machinery is reconstructed at the recombination sites has not yet been characterized [47].

Recently, a dual fork convergence model was proposed based on biochemical studies of *Xenopus* egg extracts [48–51]. In this case, even if a single fork collapses because of ICLs, the stalled replication fork remains at the lesion site until another replication fork reaches the ICL from the other side. As the incision occurs at the DNA replication termination site, reloading of the complete DNA replication machinery is not required after incision. SLX4 is involved in the selection of structure-specific endonucleases and introduces incisions in one strand of the DNA on both sides of the ICL lesion (Fig. 1c). For cleavage of ICLs, it seems that XPF-ERCC1 and SLX1 are preferentially selected, but all the components of the entire incision complex have not been identified.

## A new component that recognizes ICL lesions in mammalian cells

**Fig. 2:**
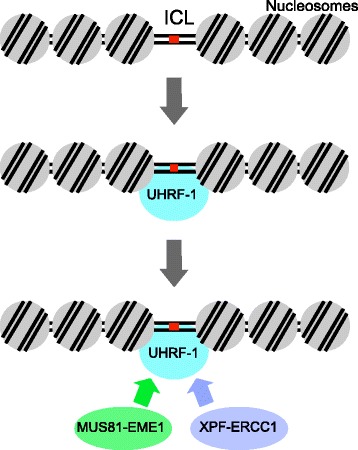
UHRF1-dependent recruitment of structure-specific endonucleases to ICLs. UHRF-1 is involved in the recruitment of FANCD2 and other DNA repair factors including XPF-ERCC1 and MUS81-EME1. Detailed mechanisms how UHRF1 recruits ICL repair factors are unclear

Recently, two groups independently discovered that a ubiquitin-like protein with both PHD and RING finger domains, UHRF1, directly recognizes ICL lesions (Fig. 2) [52, 53]. UHRF1 recognizes specific forms of histones and hemi-methylated DNA, and recruits DNMT1 [54, 55]. It is known that *UHRF1*-deficient ES cells exhibit hypersensitivity to DNA damaging agents such as ionizing radiation, UV light, *N*-methyl-*N*ʹ-nitro-*N*-nitrosoguanidine, and hydroxyurea [56]. UHRF1 strongly responds to ICLs formed by trimethyl psoralen and mitomycin C, and exhibits a weaker response to those formed by cisplatin [53]. This is because UHRF1 preferentially recognizes ICLs that cause minor distortions of the DNA helix, such as those formed by either trimethyl psoralen or mitomycin C. In contrast, ICLs formed by cisplatin cause a major distortion [2]. Both groups suggested that the likely role of UHRF1 is the recruitment of structure-specific endonucleases such as XPF-ERCC1 and MUS81-EME1 (Fig. 2) [52, 53]. However, Tian et al. argued that recruitment of nucleases is required for FA functions [52], while Liang et al. suggested that recruitment of nucleases by UHRF1 is independent of FA pathway components [53]. The mechanism of ICL recognition remains unclear and further investigation is required to precisely elucidate the mechanism.

## Perspective

Recently, many factors involved in ICL repair have been identified, particularly in mammalian cells. In quiescent eukaryotic cells from species including yeasts and higher eukaryotes, both the first and second rounds of ICL incisions are performed by NER. In contrast, not all NER factors are involved in ICL repair in S phase cells in higher eukaryotes. Instead, a variety of structure-specific endonucleases, TLS, HR, and FA pathways are required for ICL repair in S phase cells. One open question is whether ICL repair is error-free repair or not. ICL usually occurs between purine residues, however, it may also happen between pyrimidine residues. Because of such complexity, TLS bypass have no grantee to maintain error-free bypass for all ICL sites. To understand the accuracy of ICL repair requires further investigation.

In addition, very recent studies suggest that UHRF1 recognizes ICL lesions independent of the FA pathway, which is activated by recognition of stalled DNA replication forks. In ICL repair, the role of chromatin reorganization is poorly understood relative to other DNA repair pathways. One important phenomenon related to ICL repair is that NHEJ is not required for the DSB repair after ICL incision. Single DNA ends, which are produced by DSB formation at DNA replication forks, are selectively repaired by HR. In contrast, two DNA ends are predominantly repaired by NHEJ in mammalian cells. However, the dual fork incision model produces two DNA ends. If broken ends produced by dual fork incision are selectively repaired by HR, chromatin structures might be a key factor in selecting a DSB repair pathway. Certainly, many factors involved in chromatin reorganization will be identified in the future as co-factors of UHRF1 that participate in ICL repair. This will provide an engaging challenge for researchers in this field.

## Conclusions

From human genetic studies, many factors involved in ICL repair were identified. FA core complex play important roles to conduct ICL-recognition as well as DSB repair by HR. As unhooking enzymes, several structure-specific endonucleases, SLX4-SLX1, FAN1, and XPF-ERCC1, were also identified. Based on these, several models of ICL repair in S phase were proposed. In this review, we summarized the resent achievements of ICL repair. However, to discuss the entire mechanism of ICL repair, many factors are still missing. Therefore, many new factors will be discovered in the near future. In addition, understanding the molecular mechanisms of ICL repair also contribute to studies of genome instabilities and mutagenesis caused by ICL agents. Studies of ICL repair will certainly attract attention of researchers in this field for a while.

## Abbreviations

BER: base excision repair
CS: Cockayne syndrome
COFS: cerebro-oculo-facio-skeletal syndrome
DSB: double-strand break
FA: Fanconi anemia
HBOC: hereditary breast and/or ovary cancer syndrome
HR: homologous recombination
ICL: interstand crosslink
MMR: mismatch repair
NER: nucleotide excision repair
NHEJ: non-homologous end-joining
TLS: translesion DNA synthesis
TTD: trichothyodistrophy
XP: xeroderma pigmentosum
